# Gonococcal Conjunctivitis in Prepubertal Children

**DOI:** 10.1177/10775595251328692

**Published:** 2025-03-18

**Authors:** Sara Kruczek, Alexandra Dubinin, Natalie Laub

**Affiliations:** 1 Division of Child Abuse Pediatrics, 8784 Rady Children’s Hospital, San Diego, CA, USA; 2 Chadwick Center for Children & Families, 8784 Rady Children’s Hospital, San Diego, CA, USA; 3 Department of Pediatrics, University of California at San Diego School of Medicine, San Diego, CA, USA; 4 8784 University of California at San Diego School of Medicine, La Jolla, CA, USA

**Keywords:** childhood sexual abuse, sexual abuse, sexual assault, sexually transmitted infection, child abuse, childhood maltreatment

## Abstract

Conjunctivitis due to *Neisseria gonorrhoeae* is uncommon in prepubescent children. When identified in this age group, it is highly concerning for sexual contact and warrants further evaluation. This report examines 6 cases of gonococcal conjunctivitis in prepubertal children and provides updated guidance on extragenital testing for sexually transmitted infections in this population. We retrospectively reviewed cases of gonococcal conjunctivitis in prepubertal children at our institution from 2007–2022. Neonates and adolescents with this condition were excluded as were cases in which confirmatory testing was not completed. Six prepubertal children had confirmed gonococcal conjunctivitis due to *N. gonorrhoeae* from 2007–2022. Ages ranged from 8 months to 8 years. Fifty percent of cases had sexually transmitted infections at additional anatomical sites. Although rare, gonococcal conjunctivitis due to *N. gonorrhoeae* in prepubertal children should prompt further comprehensive sexually transmitted infection testing and medical evaluation. Protective agency reporting is also advised.

## Introduction

Conjunctivitis is regularly encountered in the pediatric medical setting. Young children with conjunctivitis most commonly are infected with bacteria or viruses; however, allergic etiologies exist as well (Chawla et al., 2001; Teoh & Reynolds, 2003). *Neisseria gonorrhoeae* is a less common cause of conjunctivitis outside of the neonatal or sexually active adolescent age groups. When *N. gonorrhoeae* is identified in young prepubertal children, sexual abuse must be considered (Jenny et al., 2013; Kellogg et al., 2023; Woods, 2005).

Although gonococcal conjunctivitis has been described in adult literature, the literature regarding prepubertal children with this disease mostly consists of case reports. Some of these reports theorize non-sexual modes of transmission as the cause for gonococcal conjunctivitis in prepubertal children despite incomplete assessments for the possibility of sexual abuse (Doyle, 1972, 1974; Rana & Gurung, 2021). These and other case reports focus on the utilization of culture methods as the only testing modality used to identify *N. gonorrhoeae* in prepubertal cases (Lewis et al., 1990). Although culture is still an option for testing for *N. gonorrhoeae*, the use of nucleic acid amplification testing (NAAT) has been recommended as a testing modality for this disease process in the pediatric population as well (Centers for Disease Control and Prevention, 2021).

This brief report provides an overview of extragenital testing recommendations in prepubertal children identified with gonococcal conjunctivitis outside of the neonatal period. The goal of this report is to discuss common presentations, highlight testing modalities, and show how delays in diagnosis can occur.

## Materials and Methods

### Study Population and Data Collection

The medical records of patients who presented to an urban tertiary children’s hospital from 2007 to 2022 were reviewed to identify those with a confirmed diagnosis of gonococcal conjunctivitis by *N. gonorrhoeae*. The starting date corresponds to the first patient diagnosed with gonococcal conjunctivitis since utilization of a new electronic medical record system at our institution. Our goal was to identify prepubertal children with gonococcal conjunctivitis as this population cannot consent to sexual activity in the United States. Neonates and sexually active adolescents were excluded from this study as their acquisitions of gonococcal conjunctivitis were likely due to vertical transmission at birth or known sexual activity, respectively. Data regarding demographic information, presenting symptomatology, number of medical encounters, and clinical course were collected. This report was deemed exempt from human subject research by the Institutional Review Board at our institution and conformed to the requirements of the US Health Insurance Portability and Accountability Act of 1996.

### Laboratory Methods

Culture: Discharge coming from the lid margin of each eye was collected for culture in most cases. These cultures were inoculated on Chocolate agar and Modified Thayer-Martin agar plates which were then incubated at 35 degrees C in 5–7% CO_2_. Plates were first read at approximately 18–24 hours after setup and checked again at 48 and 72 hours of incubation. When growth was present, a gram stain was performed to look for gram negative diplococci and oxidase testing was performed. Isolates were then run on the MALDI-TOF (Bruker Corp, Billerica, MA) for identification.

Nucleic Acid Amplification Testing (NAAT): Testing involved samples collected from the throat, rectum, urine, and lid margins for the majority of cases. Two cases did not have rectal swabs collected and one did not have urine collected. It is unclear why these samples were not collected as it is standard practice to do so at our institution. Our hospital system uses Aptima Combo II (Hologic, San Diego, CA) for testing. As per the package insert, the Aptima Combo II is a target amplification nucleic acid probe test that utilizes target capture for detection and differentiation of ribosomal RNA from *Chlamydia trachomatis* and/or *N. gonorrhoeae*. Confirmation was completed via alternate target testing of the samples collected or with newly obtained samples.

## Results

**Figure 1.**
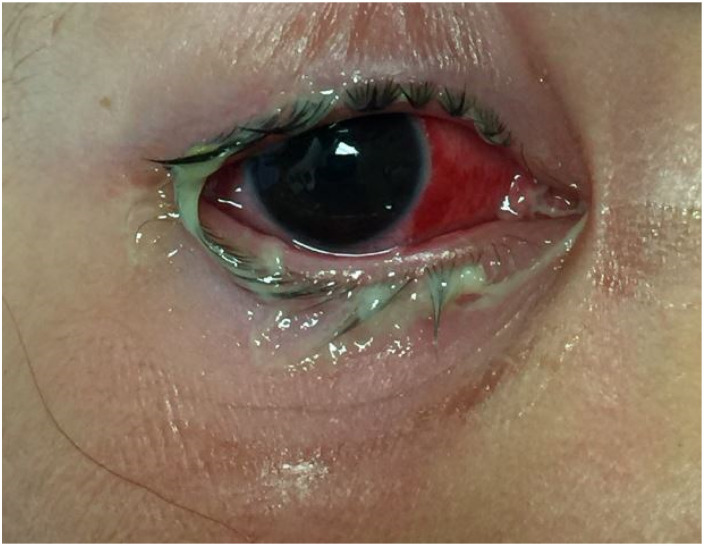
6-year-old female with right eye chemosis, 2+ subconjunctival hemorrhage nasally, and mucopurulent discharge diagnosed with gonococcal conjunctivitis.

**Figure 2.**
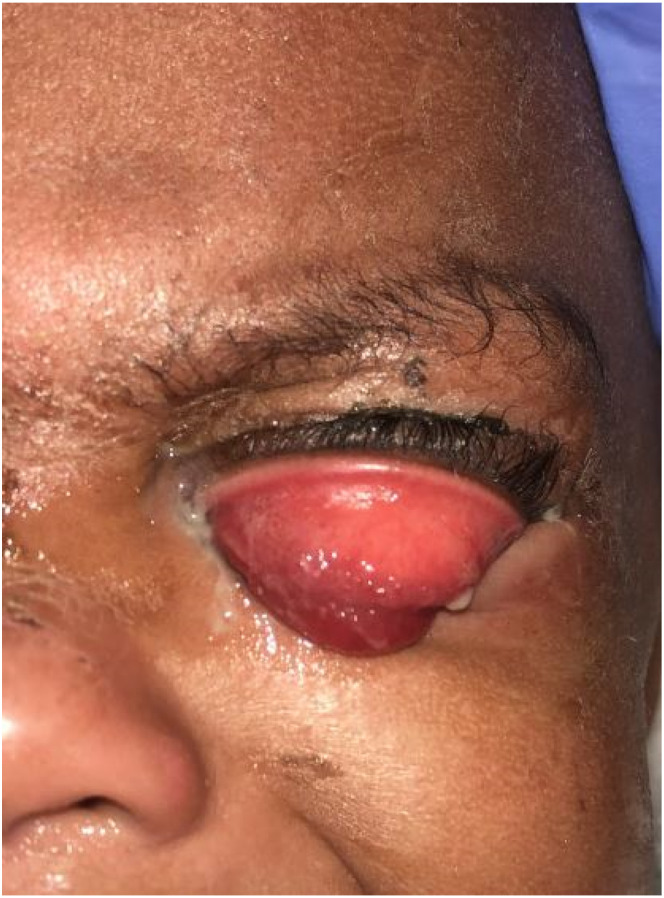
15-month-old female with bilateral periorbital swelling, purulent ocular discharge and an everted left upper eyelid diagnosed with gonococcal conjunctivitis.

A total of six young children outside of the neonatal period were diagnosed with gonococcal conjunctivitis within our tertiary children’s hospital network from 2007 – 2022. Of these, 5 (83%) were female. The ages of these patients ranged from 8 months to 8 years. Four (67%) of the six children had been evaluated by a medical provider at least once with symptoms of profound conjunctivitis (eye redness, mucopurulent discharge, and eyelid swelling) prior to any testing which confirmed the presence of *N. gonorrhoeae *(Figure 1 and Figure 2). Four (67%) of the children underwent imaging of the orbits, three of which had evidence of pre-septal cellulitis. Most of the children, 5 (83%), were admitted to the hospital for intravenous antibiotics (ceftriaxone and clindamycin) for treatment of pre-septal cellulitis. Once *N. gonorrhoeae* was identified, each case received azithromycin to cover for co-infection with *C. trachomatis* as well. The antibiotic regimens used to treat gonococcal conjunctivitis in these children followed standard treatment guidelines for age and weight (Kimberlin, 2021). The only child that was not admitted to the hospital was seen in the Child Abuse Pediatrics clinic where he received an intramuscular dose of ceftriaxone and a dose of azithromycin to cover for co-infection with *C. trachomatis*. Of the children in this study, 3 (50%) had evidence of sexually transmitted infections in other anatomic sites including the rectum, vagina, and throat (Table 1). One (17%) child had *N. gonorrhoeae* identified in the throat which may represent contiguous spread from one site to the other. No child in this study tested positive for human immunodeficiency virus (HIV), syphilis, hepatitis B, or hepatitis C. Of the two children old enough to disclose sexual abuse, neither child provided a disclosure. All children in this series had resolution of symptoms after appropriate treatment.

**Table 1.** Characteristics of Population and Testing Modalities.

Case	Age	Sex	Symptoms	Ocular testing (by medical encounter)	Additional sites*
1	15 months	F	Purulent discharge periorbital swelling URI symptoms	Eye discharge culture: No growth^a^ (encounter 2) Periorbital skin swab NAAT: + NG (encounter 3)	Throat GC/CT NAAT: Negative Rectal GC/CT NAAT: Negative Urine GC/CT NAAT: Negative HIV/Syphilis/HBV/HCV: Negative
2	15 months	F	Purulent discharge periorbital swelling pseudo membrane conjunctival injection Fever	Eye discharge culture #1: + NG (encounter 1) Eye discharge culture #2: + NG (encounter 2) Periorbital skin swab NAAT: + NG (encounter 3)	Throat GC/CT NAAT: +NG Rectal GC/CT NAAT: Negative Urine GC/CT NAAT: Negative HIV/Syphilis/HBV/HCV: Negative
3	8 years	M	Purulent discharge eyelid swelling Conjunctival injection	Periorbital skin swab NAAT: + NG (encounter 2)	Throat GC/CT NAAT: Negative Rectal GC/CT NAAT: Negative Urine GC/CT NAAT: Negative HIV/Syphilis/HBV/HCV: Negative
4	6 years	F	Purulent discharge periorbital swelling SCH	Eye discharge culture: + NG (encounter 1) Periorbital skin swab NAAT: + NG (encounter 2)	Throat GC/CT NAAT: + NG Rectal GC/CT NAAT: + NG Urine GC/CT NAAT: + NG HIV/Syphilis/HBV/HCV: Negative
5	8 months	F	Purulent discharge periorbital swelling conjunctival injection fever	Eye discharge culture: + NG (encounter 2)	Throat GC/CT NAAT: Negative Urine GC/CT NAAT: Negative HIV/Syphilis/HBV/HCV: Negative
6	2 years	F	Purulent discharge periorbital swelling conjunctival injection URI symptoms Fever	Eye discharge culture: + NG (encounter 3)	Throat GC/CT NAAT: + CT HIV/Syphilis/HBV/HCV: Negative

NG, *Neisseria gonorrhoeae;* CT, *Chlamydia trachomatis;* NAAT, RNA Nucleic Acid Amplification Test; SCH, Subconjunctival hemorrhage; HIV, Human immunodeficiency virus; HBV, Hepatitis B virus; HCV, Hepatitis C virus.

*Testing completed at time of ocular testing or at subsequent medical encounter.

^a^Treated with ceftriaxone prior to collection of eye discharge culture.

## Discussion

Gonococcal conjunctivitis is infrequently identified in the prepubertal population, outside of the neonatal period. Although this infection is uncommon, it can result in profound medical consequences if left untreated to include corneal ulceration, globe rupture, scarring, and visual impairment leading to blindness (Bodurtha Smith et al., 2017; Lopes et al., 2023; McAnena et al., 2015). For these reasons, timely diagnosis and treatment is important.

Our brief report highlights 6 cases of prepubertal children who presented with mucopurulent eye discharge and eyelid swelling. There was a delay in identifying *N. gonorrhoeae* in 4 of the children as front-line clinicians did not complete any testing until the second or third medical visit for conjunctivitis (Table 1). This is likely due to availability bias, meaning clinicians judge the likelihood of a diagnosis on similar examples seen in clinical practice (Saposnik et al., 2016). Thus, clinicians are unlikely to think about sexually transmitted diseases as a cause for infection in a prepubertal child. Because children who have been sexually abused frequently don’t disclose the abuse they’ve endured (Goodman-Brown et al., 2003; Hebert et al., 2009; Paine & Hansen, 2002; Sjöberg & Lindblad, 2002; Wallis & Woodworth, 2020), there is no historical information to alert the clinician to order the testing needed to confirm the diagnosis.

Current standard of care for testing extragenital sites (rectum and throat) for *N. gonorrhoeae* and *C. trachomatis* in children is to use a Clinical Laboratory Improvement Amendments (CLIA)-validated, Food and Drug Administration (FDA)-cleared NAAT with confirmation or use culture (Centers for Disease Control and Prevention, 2021). NAATs can be preferable to culture because culture performance is dependent upon the sample collection technique and a negative culture result does not rule out infection (Black et al., 2009; Qin & Melvin, 2020). NAATs can also be useful in cases where children have received a dose of antibiotics prior to testing and have a negative culture, as seen in case 1 of this study. There is no reason to believe that NAAT performance characteristics would vary based on site tested. For the reasons listed above, our best recommendation for ocular testing is to use an FDA-cleared NAAT when testing ocular discharge. If the results are positive, confirmatory testing must be completed. Expert consultation should be utilized to help with correct interpretation of results if non-FDA approved testing modalities are used in extragenital site testing of prepubertal children. When *N. gonorrhoeae* is isolated from the genitals, rectum, or pharynx of prepubertal children outside of the neonatal period, it is diagnostic of sexual contact (Hammerschlag & Guillén, 2010; Kellogg et al., 2023). Less is known about the mode of transmission for ocular involvement of *N. gonorrhoeae* in prepubertal children. Contiguous spread from other anatomic sites cannot be ruled out as a cause for ocular involvement. Other non-sexual modes of transmission should only be considered after complete assessment has identified isolated ocular gonorrhoeae in absence of other evidence of sexual abuse such as physical exam findings consistent with sexual abuse, patient disclosures, or sexually transmitted infections of the anogenital sites.

Medical providers who evaluate children should keep gonococcal conjunctivitis on the differential diagnosis when encountering children with symptoms of mucopurulent eye discharge. When *N. gonorrhoeae* is identified as the cause of conjunctivitis in prepubertal children, comprehensive sexually transmitted infection testing is recommended as well as a medical evaluation by a clinician trained in child sexual abuse exams (Jenny et al., 2013; Uprety & Cárdenas, 2019). A report to child protective services and law enforcement is advised when child sexual abuse is suspected.
